# Supplementary material for: Characterization and Expression Analysis of the ALOG Gene Family in Rice (Oryza sativa L.)
Source: Plants (Basel). 2025 Apr 14;14(8):1208. doi: 10.3390/plants14081208 (PMC12030751; doi:10.3390/plants14081208)
Supplement: Supplementary file 1 [file plants-14-01208-s001.zip › Supplementary files/supplymentary figureS1-S6_20250404_revised.pdf]

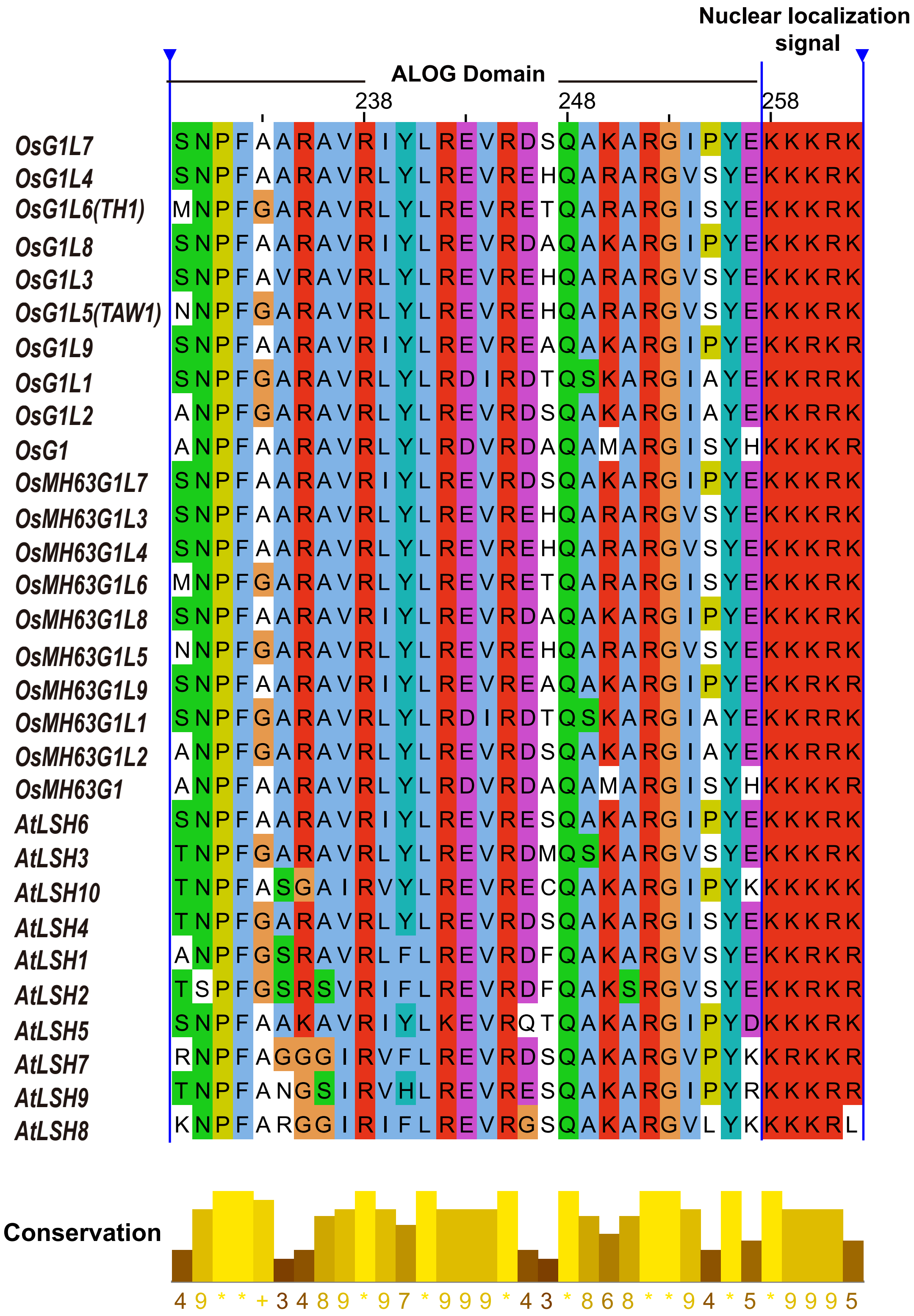

Figure S1. Conserved ALOG and nuclear localization signal domains of 30 ALOG family proteins.

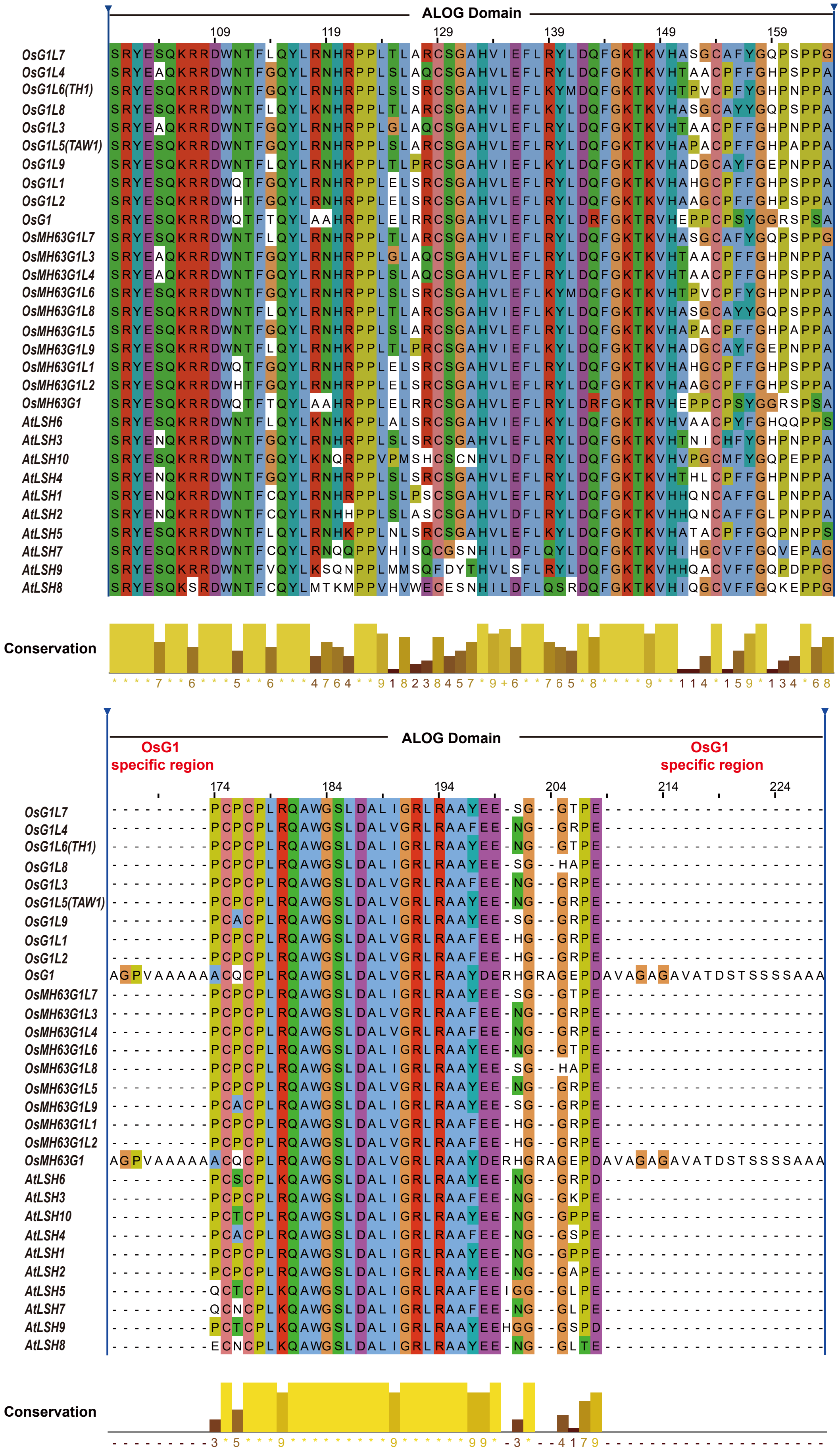

Figure S2. The specific regions of OsG1 and OsMH63G1 in the ALOG domain.

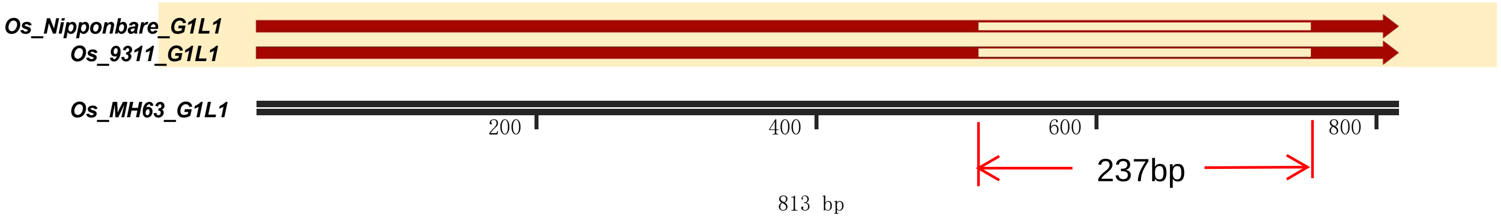

Figure S3. The 237bp insertion in the coding region of the *OsMH63G1L1*.

Motif1 50

Motif2 41

Motif3 41

Motif4 29

Motif5 16

Motif6 20

Motif7 41

Motif8 19

Motif9 6

Motif10 17

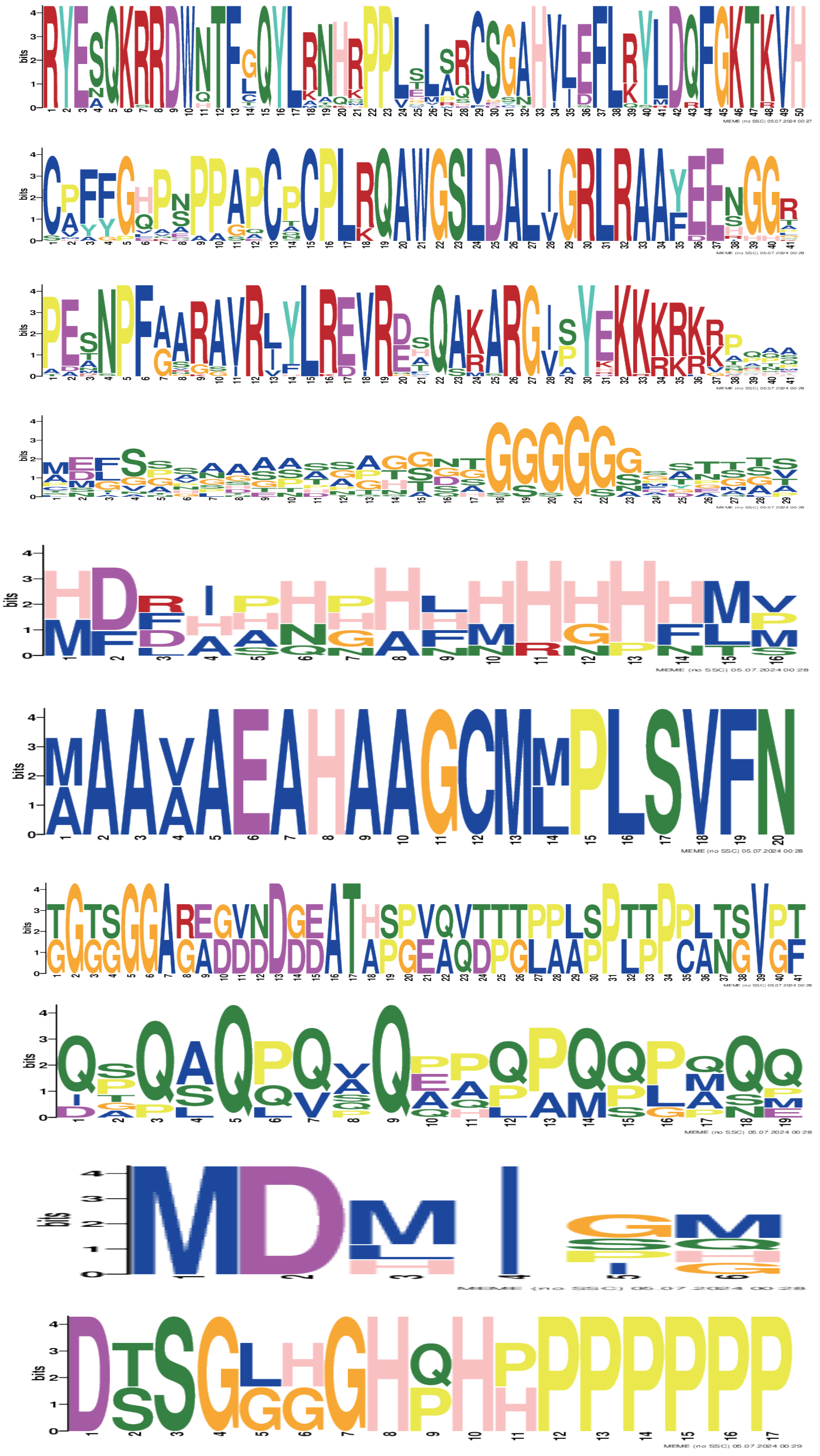

Figure S4. The ten motifs of ALOG proteins. The numbers below represent the counts of amino acid residues.

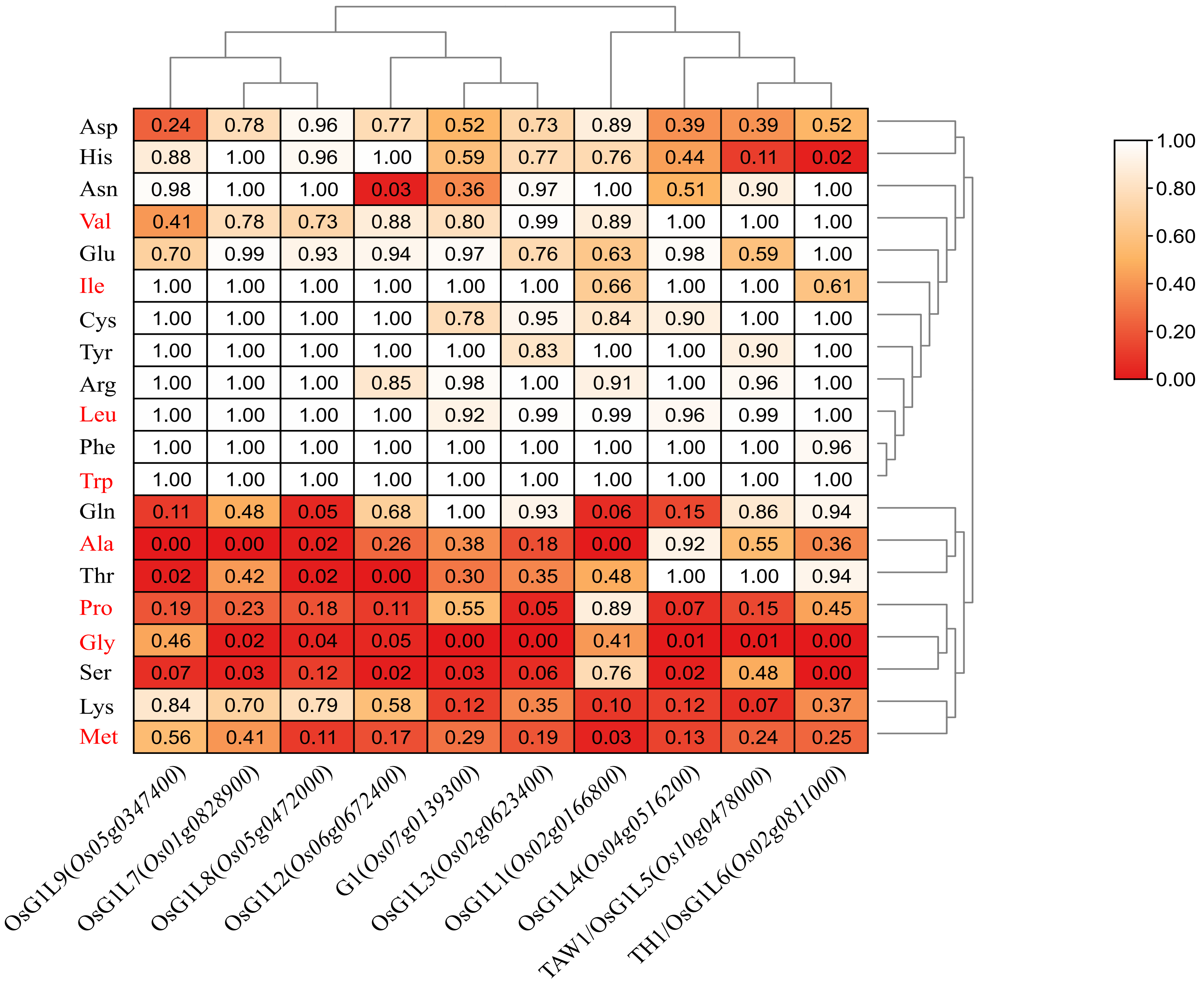

Figure S5. Enrichment analysis of residues in IDRs. The red font represent non-polar residues. The numbers within the rectangles are P-values. Hierarchical clustering was used to classify the enrichment characteristics of each amino acid in IDRs.

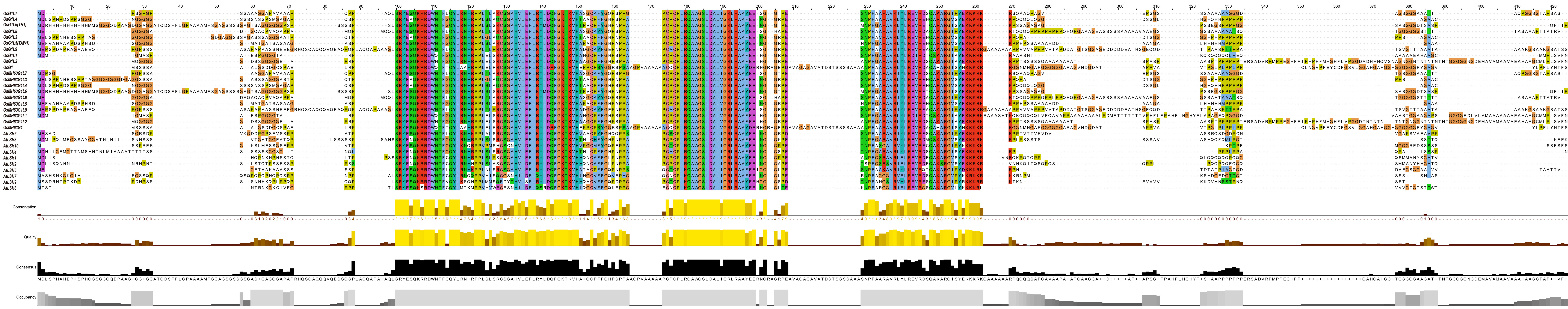

Figure S6 The complete amino acid sequence alignment of 30 ALOG members
